# Methamphetamine Overdose and Hyperthermia in a 41-Year-Old Man Found Unresponsive

**DOI:** 10.7759/cureus.95950

**Published:** 2025-11-02

**Authors:** Christian G Frey, Bryan Wilson, Hailey P Hoffmann, John R Zatarain, Chiemela Ubani, Krishna K Paul, Dietrich V Jehle

**Affiliations:** 1 Department of Emergency Medicine, University of Texas Medical Branch at Galveston, Galveston, USA; 2 Pharmacy Department, University of Texas Medical Branch at Galveston, Galveston, USA

**Keywords:** amphetamine, benzodiazepines, cooling strategies, heat related illness, hyperthermia, methamphetamine, toxicity

## Abstract

This case examines the death of a 41-year-old man due to acute methamphetamine toxicity and severe hyperthermia. The patient was found unresponsive with a pulse and spontaneous respirations along a beach in Southeast Texas. Local weather at noon in late August was reported as 92°F (33.3°C) with 60% humidity. On arrival at the emergency department, he was unresponsive with agonal respirations and no palpable pulse. His core body temperature was 108.6°F (42.6°C) measured rectally, one of the highest temperatures recorded in a patient with a methamphetamine overdose who had a pulse and was breathing. Despite aggressive resuscitation and active cooling for 30 minutes, he was pronounced dead with a final temperature of 104.3°F (40.2°C). Postmortem toxicology from femoral blood revealed a methamphetamine concentration of 680 ng/mL and an amphetamine concentration of 55 ng/mL. This case provides an opportunity to discuss the multiple mechanisms through which stimulants contribute to hyperthermia as well as potential treatment strategies.

## Introduction

Methamphetamine is a powerful central nervous system stimulant originally prepared for clinical use for the treatment of obesity, narcolepsy, and attention deficit disorder [1]. Methamphetamine is currently rarely prescribed due to its abuse potential. Additionally, recreational use and addiction with methamphetamine and racemic amphetamine are common. These amphetamines exert their effects by increasing synaptic concentrations of monoamines - particularly dopamine, norepinephrine, and serotonin - leading to heightened alertness, increased energy, and euphoria [2]. In methamphetamine overdose, an excess of monoamine activity can result in severe cardiovascular, neurological, and thermal dysregulation [3]. Clinically, this presents as tachycardia, hypertension, agitation, delirium, seizures, and hyperthermia [4].

Severe hyperthermia, defined as a core body temperature exceeding 104°F (40°C), is of particular concern if not promptly treated [5]. Through multiple mechanisms, methamphetamine inhibits the body’s thermoregulatory mechanisms and can lead to a rapid rise in temperature. When the core temperature exceeds 108°F (42.2°C), the risk of death becomes extremely high due to the irreversible damage to cellular structures and vital organs [5]. In such cases, the combination of methamphetamine-induced hyperthermia and an already elevated ambient temperature, such as the conditions experienced in Southeast Texas can quickly become lethal.

This case report details the death of a 41-year-old man due to hyperthermia secondary to methamphetamine toxicity and warm ambient temperatures and correlates it with post-mortem blood levels of methamphetamine and amphetamine. This case represents one of the highest temperatures recorded in an individual with methamphetamine toxicity and a pulse. The case provides an opportunity to review the pathophysiologic mechanisms underlying hyperthermia in methamphetamine toxicity, highlight the importance of rapid and aggressive intervention, and to review potential cooling strategies.

## Case presentation

Patient information

The patient was a 41-year-old man, weighing 249 pounds (113 kg). He had no known medical history. A call was made by a third party to report the incident, and emergency medical services (EMS) arrived five minutes after being dispatched. The patient was found unresponsive by EMS near the beach in Southeast Texas. Local weather conditions were sunny with a temperature of 92°F (33.3°C) and 60% humidity. The patient had a pulse rate of 88, blood pressure (BP) 78/palp, oxygen saturation (O_2_ sat) of 69%, and was breathing spontaneously at 40 breaths per minute on EMS arrival at the scene. A left antecubital intravenous (IV) was attempted but unsuccessful. An 18-gauge catheter was successfully placed in the left hand. The patient felt extremely warm to the touch and ice packs were placed on the groin area and armpits. Medics were on scene for approximately 10 minutes. Glucose level was 266 mg/dL. A non-breather mask was placed on the patient at 25 L/min. He was transported to the emergency department, lights and sirens over seven minutes. Repeat vital signs en route demonstrated blood pressure at 62/74 mmHg, respiratory rate of 39 breaths per minute, and O_2_ sat improved to 78%. A 12-lead ECG in the ambulance showed sinus tachycardia at 142 bpm with diffuse non-specific ST-T wave changes. One liter of normal saline was infused en route to the emergency department (Table 1).

**Table 1:** Clinical Timeline of Events – Methamphetamine Overdose with Hyperthermia BP: Blood pressure; SpO_^2^_: peripheral oxygen saturation; RR: respiratory rate; NRB: non-rebreather bag; CPR: cardiopulmonary resuscitation; ACLS: advanced cardiovascular life support; IO: intraosseous; PEA: pulseless electrical activity; NS: normal saline; US: ultrasound; ME office: medical examiner's office.

Pre-Hospital (Scene and EMS)		
Time	Event	Labs and Vitals
~11:55 AM	Patient (41-year-old male, 249 lb) found unresponsive near a beach in Southeast Texas. Weather was 92°F (33.3°C), 60% humidity. Patient warm to touch. Unsuccessful IV in left antecubital; 18-gauge IV placed in left hand. Ice packs applied to groin/axilla. Non-rebreather mask at 25 L/min applied.	Initial vitals: Pulse 88 BP 78/palp, SpO₂ 69%, RR 40/min, Glucose 266 mg/dL
~12:00 PM	12-lead ECG: sinus tachycardia 140s, diffuse ST-T changes.	
~12:02 PM	Cooling treatment initiated.	
~12:03 PM	Oxygen via NRB continued.	
~12:11 PM	1000 cc NS bolus given en route.	Repeat vitals: BP 62/74 SpO₂78% RR 39 ECG sinus tachycardia 142 bpm
Emergency Department (Arrival to Death)		
Time	Event	Labs and Vitals
12:14 PM	Arrival at ED. Status: Unresponsive, hot to touch, breathing at 6/min. Interventions: CPR and ACLS initiated. Intubation with 8.0 tube (confirmed). IO access (humeral head, anterior tibia). Ice packs applied (neck, axilla, groin); evaporative cooling with fans/mist; cooling blanket behind pt. IV fluids (cold saline). 7 amps epinephrine, 3 amps bicarbonate, and naloxone administered during code. Total fluids in ED: 2 L (3 L overall). Clinical course: Ventricular fibrillation → shocked (200J) → transient return of circulation (heart rate 150). Subsequent PEA (multiple US - no cardiac activity)→ asystole → CPR/ACLS continued. Despite 35 minutes of ACLS, no cardiac activity on ultrasound.	Vitals: No palpable pulse. Pupils fixed/dilated (4 mm). Rectal temperature (confirmed with temp sensing foley): 108.6°F (42.6°C). Temperature dropped gradually to 104.3°F.
Post-Mortem		
Time	Event	Labs and Vitals
3:09 PM	External Examination Findings: No significant traumatic injuries. Small abrasion on the left lower leg. Evidence of resuscitative efforts (abrasions, IO/IV lines, endotracheal tube, ECG pads, Foley catheter). Cause of Death (Galveston County ME Office): Primary: Toxic effects of methamphetamine. Contributory: Hyperthermia/heat stroke. Manner: Accident.	Toxicology results: Methamphetamine: 680 ng/mL (femoral blood). Amphetamine: 55 ng/mL. Urine: presumptive positive for fentanyl metabolite.

Clinical findings

Upon arrival at the emergency department, the patient was breathing at six breaths/min and was still unresponsive. The pulse was no longer palpable, and the patient became apneic. Pupils were fixed and dilated to 4 cm. Physical exam showed no signs of trauma outside of a small abrasion on the lower left leg. Upon the patient's arrival at the emergency department, his severe hyperthermia, unresponsiveness, and core body temperature of 108.6°F (42.6°C) signaled a dire prognosis. The initial treatment approach focused on stabilizing the patient’s airway, breathing, and circulation, which involved immediate intubation to secure the airway and mechanical ventilation to manage respiratory function. Correct intubation with an 8.0 tube was confirmed by auscultation and capnography. Advanced cardiac life support (ACLS) protocols were initiated, including support of cardiac output and addressing the ventricular fibrillation that occurred during resuscitation efforts. The patient was initially treated with epinephrine, bicarb, cardiopulmonary resuscitation (CPR), intermittent ultrasound checks, humeral head and tibial intraosseous (IO) lines, and cooled intravenous (IV) fluids. After two rounds of CPR, the patient required countershock once for ventricular fibrillation with return of pulses. The next pulse check showed the pulse at 150 BPM and sinus tachycardia. A temperature measuring foley catheter was placed and heart rate decreased to 60 BPM. The rectal temperature was measured at 108.6°F (42.6°C) at this time, indicating severe hyperthermia. Evaporative cooling was initiated with fans and cool mist directed at the anterior chest, while cooling blankets were applied posteriorly and ice packs were placed at the neck, groin, and axilla in accordance with ACLS protocol. Pulses were re-lost, and CPR was initiated for rhythms of pulseless electrical activity (PEA) and subsequent asystole. Multiple bedside ultrasound (US) confirmed the absence of cardiac activity. The patient received seven ampules of epinephrine, three ampules of bicarbonate, one ampule of Narcan in addition to two liters of fluid in the emergency department. The next pulse check revealed no pulse with asystole on the monitor and no cardiac activity on bedside US. Throughout the resuscitation, there was no shivering observed in the emergency department. Despite aggressive resuscitative efforts and active cooling, the patient could not be revived. His body temperature dropped steadily by approximately 4°F over the 35 minutes of resuscitation in the emergency department. The final rectal temperature was measured at 104.3°F (40.2°C), and this was confirmed with Foley temperature monitoring. Emergency department labs, other than those ordered by the coroner's office, were discontinued after the patient was declared dead (Table 1).

Toxicology report

Postmortem toxicology from femoral blood revealed a methamphetamine concentration of 680 ng/mL and an amphetamine concentration of 55 ng/mL. These findings indicate a high level of stimulant use, well within the range associated with fatal outcomes (Table 1).

## Discussion

This is one of the highest temperatures recorded in a patient with a methamphetamine overdose who had a pulse and was breathing. In this case of a 41-year-old man who succumbed to acute methamphetamine toxicity and severe hyperthermia, the emergency treatment team faced a critical situation requiring rapid and precise intervention. Active cooling measures were critical; cooling blankets were applied, along with ice packs to the neck, groin, and axillae to reduce core body temperature. Additionally, evaporative cooling techniques were employed. IV fluids were also administered to help lower the patient’s temperature internally. Despite these aggressive and ACLS interventions, the patient’s condition did not improve, and he could not be revived. The severity of his hyperthermia, combined with the toxic effects of methamphetamine, overwhelmed the available treatment options, ultimately leading to his death.

Methamphetamine, a potent stimulant, exerts its effects by increasing the release of monoamines like dopamine, norepinephrine, and serotonin [2]. At the cellular level, methamphetamine leads to increased neuronal activity and excessive neurotransmitter release, which can cause significant oxidative stress and excitotoxicity [6]. This hyperactivity can disrupt cellular functions, leading to severe cardiovascular, neuropsychiatric, and thermoregulatory disturbances [7]. When taken in excess, methamphetamine can overwhelm the body’s ability to maintain homeostasis, resulting in potentially fatal outcomes, especially when combined with external factors such as high ambient temperatures. Treatment of methamphetamine toxicity emphasizes aggressive resuscitation and cooling to manage hyperthermia [7,8]. Sedation is often used to treat agitation, seizures, and minimize heat generation from shivering, but these symptoms were not present in this case.

Determining the exact lethal dose for 50% of the population (LD50) of methamphetamine in humans is challenging due to individual differences in tolerance, purity of the substance, and other variables [7]. The LD50 in animal studies has been estimated to be between 55 and 57 mg/kg for rats and mice, although intravenous use can lower this threshold significantly [7,9]. Levels of methamphetamine in the blood have also been shown to increase postmortem due to redistribution, making it more difficult to pinpoint the LD50 [10]. However, methamphetamine concentrations in the blood exceeding 200 ng/mL are often associated with fatal outcomes, particularly when complicated by hyperthermia or other factors [7,11].

Hyperthermia induced by methamphetamine use is particularly dangerous because the drug impairs the body’s ability to regulate temperature [12,13]. Methamphetamine use affects the hypothalamus, which is responsible for thermoregulation, and disrupts the body’s heat dissipation mechanisms [13]. The increase in dopamine and norepinephrine exacerbates peripheral vasoconstriction, limiting heat loss through the skin [13]. Furthermore, the drug-induced hypermetabolic state increases heat production, pushing the body’s temperature to dangerous levels [14]. Methamphetamine’s effects on thermoregulation are particularly problematic as ambient temperatures rise. Previous literature has demonstrated an increase in emergency department visits for opioid, cocaine, and amphetamine overdoses beginning at mild ambient temperatures above 60°F (15.5°C) [15]. Additionally, deaths related to cocaine have been shown to increase as peak daily ambient temperatures approach 75°F (24°C) with a prominent increase as temperatures pass above 88°F (31.1°C) [16,17].

The combination of methamphetamine’s effects on thermoregulation and heat stress from ambient conditions can lead to severe hyperthermia, resulting in multiorgan dysfunction and eventually death. Cellular proteins begin to denature, and enzymatic functions are impaired as core body temperature rises above 104°F (40°C). When temperatures exceed 108°F (42.2°C), irreversible damage to vital organs, as seen in this patient, occurs [18-20]. However, this level of temperature exposure has been shown to be exponentially dependent on exposure time as well [19-20]. Rodent models show that the LD50 (lethal dose 50) in regard to time of temperature exposure to 42.5°C is about 25 minutes without any previously acquired resistance to heating or thermotolerance [21]. There is limited information on the exact LD50 of human core body temperature due to this relationship with exposure time. However, due to the lack of thermotolerance development at 42.5°C in mammalian cell lines shown in the Raaphorst et al. study [21] on hyperthermia in cancer treatments, it can be reasonably concluded that a core temperature of 42.5°C is generally unsustainable with life [22]. There has also been significant differences shown in rodent survival when exposed to 42.25°C versus 42.5°C [5]. These findings underscore the critical threshold at which increases in core body temperature can drastically reduce survival time, highlighting the extreme vulnerability of biological systems to hyperthermia at temperatures near 42.5°C. Therefore, prioritization of hyperthermia management is of utmost importance in patients with these temperatures. In addition, despite some differences in pathophysiology, research on exertional heat stroke in athletes can provide some relevant human data. This literature supports a mortality rate above 25% and an increase in morbidity and mortality with increased exposure to temperature and time. Additionally, athletes with exertional heatstroke who go unrecognized or are not cooled quickly and have more than 60 min of temperature elevation above 40.5°C (105°F) tend to have increased morbidity and mortality [22]. A documented case of methamphetamine intoxication in San Antonio, Texas, involved an individual found deceased and pulseless on asphalt on a day with an ambient temperature of 106°F. Postmortem toxicology revealed methamphetamine concentrations slightly above therapeutic but below established toxic ranges [23]. The individual’s core body temperature measured 126°F at autopsy. Following cardiopulmonary arrest, body temperature trends toward equilibrium with environmental and surface conditions. Asphalt, due to its dark color and high thermal mass, readily absorbs solar radiation and retains heat for prolonged periods. At ambient temperatures of 80-90°F, asphalt can reach approximately 125°F, and when air temperatures exceed 100°F, surface temperatures may surpass 150°F. Prolonged contact with such surfaces can exacerbate hyperthermia and accelerate postmortem temperature elevation.

Methamphetamine use can further predispose individuals to hyperthermia through increased metabolic rate, peripheral vasoconstriction, and impaired thermoregulatory mechanisms. These effects may be compounded by environmental heat exposure, reduced behavioral capacity to seek shade or hydration, and concomitant dehydration. Epidemiologic data underscore this interaction: the 2023 Heat Death Report from the Maricopa County Department of Public Health documented 645 heat-related deaths, a 52% increase from the prior year. From 1999 to 2023, heat-related mortality in the United States rose by 117%, with substance use present in 65% of cases; methamphetamine was detected in 78% of those [24]. The proportion of heat-related deaths linked to substance use increased from 33% in 2014 to 65% in 2023. Demographically, more than three-quarters of decedents were men, two-thirds were aged ≥50 years, 45% were experiencing homelessness, and 14% had a history of schizophrenia. While advanced age remains a recognized risk factor, the highest rates of mortality were observed among individuals aged 35-49 and 58-64 years [24].

These findings highlight the synergistic lethality of stimulant intoxication and extreme environmental heat, particularly among socially vulnerable populations. The presented case exemplifies the potential for fatal hyperthermia even in the absence of methamphetamine concentrations within traditionally defined toxic ranges.

Given the short window to intervene and the typical delays in arriving for medical care, rapid identification of hyperthermia and aggressive cooling are a priority to prevent morbidity and mortality in patients with methamphetamine toxicity. In the severely hyperthermic patients, it is possible that their elevated temperature is a more immediate life threat than their airway, breathing, or circulation. The best option for cooling a patient will depend on the resources available and the specific clinical circumstance. Examples of specific options are discussed below. Regardless of the method chosen, the authors encourage a preplanned approach and protocol.

There is a large variety of specific cooling methods described in the literature. Frequently discussed methods include the application of ice packs to the neck, groin, and axillae. Evaporative cooling through air flow and misting is another common technique. When available, cold intravenous fluids are also a consideration. Numerous commercial devices in the form of cooling blankets or garments, ice sheets, reflective blankets, or hand cooling devices may be available in healthcare facilities. In severe cases, more invasive methods like gastric, bladder, or peritoneal lavage with cold fluids have been suggested.

Despite the multitude of options, a recent meta-analysis supports water immersion as the most rapid non-invasive cooling strategy regardless of whether the water was iced, cold, or temperate [23]. Immersion methods cooled patients 0.27 to 0.36°F/min (0.15 to 0.2°C/min) while non-immersion methods cooled patients at 0.09°/min (0.05°C/min). The logistics of water immersion can be challenging in the hospital setting with a critically ill patient, but the improvised use of body bags and tarps has been demonstrated as feasible [24,25].

Along with active methods of cooling, benzodiazepines or other sedatives to reduce agitation and psychomotor activity are an important aspect of treating the patient’s hyperthermia. Antipsychotics should be avoided due to their risk of anticholinergic syndrome and their lack of a direct effect on the patient’s increased sympathetic tone. Acetaminophen and other antipyretics have no role in the treatment of hyperthermia due to methamphetamine toxicity.

These interventions, combined with aggressive resuscitation and supportive care, are critical in managing hyperthermia and mitigating the effects of methamphetamine toxicity. However, even with optimal treatment, the patient may have already sustained irreversible damage and experienced long-term morbidity or mortality. This emphasizes the need for rapid intervention to maximize the patient’s prognosis.

## Conclusions

This case underscores that life-threatening hyperthermia can occur with methamphetamine intoxication even in moderate ambient conditions (92°F (33.3°C), 60% humidity). Despite a rapid initiation of aggressive cooling in the emergency department, the patient succumbed, highlighting how unavoidable delays in locating and transporting patients can critically limit outcomes. Recognizing the degree of hyperthermia in the field and early initiation of immersion and ice bath/ice-filled body bag in the field could have potentially led to more rapid cooling, despite the presence of a uniformly fatal temperature in this patient. The markedly elevated initial temperature (108.6°F (42.6°C)) in conjunction with environmental heat and postmortem toxicology findings (femoral blood methamphetamine 680 ng/mL, amphetamine 55 ng/mL) reinforces the role of methamphetamine in lethal hyperthermia. This case adds to the growing evidence that both environmental and pharmacologic factors must be considered in the rapid identification and management of heat stroke in at-risk individuals.
